# Does General and Specific Traits of Personality Predict Students' Academic Performance?

**DOI:** 10.1155/2022/9422299

**Published:** 2022-01-04

**Authors:** Mohamed Jaber, Basim Al-Samarrai, Afraa Salah, Sudhir Rama Varma, Mohmed Isaqali Karobari, Anand Marya

**Affiliations:** ^1^College of Dentistry, Department of Clinical Sciences, Ajman University, Ajman, UAE; ^2^Center for Medical and Bioallied Health Sciences Research, Ajman University, Ajman, UAE; ^3^Conservative Dentistry Unit, School of Dental Sciences, Universiti Sains Malaysia, Kubang Kerian, 16150 Kota Bharu, Kelantan, Malaysia; ^4^Department of Conservative Dentistry & Endodontics, Saveetha Dental College & Hospitals, Saveetha Institute of Medical and Technical Sciences University, Chennai, 600077 Tamil Nadu, India; ^5^Department of Orthodontics, Faculty of Dentistry, University of Puthisastra, Phnom Penh, Cambodia; ^6^Department of Orthodontics, Saveetha Dental College & Hospitals, Saveetha Institute of Medical and Technical Sciences University, Chennai, 600077 Tamil Nadu, India

## Abstract

**Methods:**

The study includes all fifth-year dental students registered at the College of Dentistry, Ajman University, in 2019/2020. One hundred and seventy students were invited to complete personality and performance measures using the Big Five Inventory (BFI) scale; the weighted grade point average (GPA) was used to assess students' academic performance.

**Results:**

Of the 170 participants, 60% were female and 40% were male. Participants ranged in age from twenty-four to twenty-seven years, with an average age of twenty-four years. There was a relationship between personality scores obtained for the students and their subsequent academic performance. The broad conscientiousness, competence, achievement, and dutifulness predicted academic and clinical success. The prediction accuracy of conscientiousness was improved by the inclusion of dutifulness, self-discipline, and deliberation.

**Conclusion:**

This study confirms that the students' personality profile is a substantial predictor of academic performance and likely to help select future intakes of students, although a prospective study would be required for a definite answer to this question.

## 1. Introduction

Traditionally, dental students have been selected based on their secondary school academic performance, although intellectual ability only accounts for about 35% of the variance in performance [1]. Academic institutions vary in the cognitive and noncognitive measures used in the selection processes.

Numerous studies have investigated selection measures in medical and dental education and provided evidence supporting the role of personality in predicting success [2–4]. Chamberlain et al. [2] found that conscientiousness, neuroticism, and agreeableness variables predicted success criteria to various degrees across the study years of dental school. Likewise, Poole et al. [3], who studied 373 Canadian dental students, found that the conscientiousness variable predicted clinical and academic performance while openness to experience predicted academic performance. Stacey and Kurunathan [4] reported that the conscientiousness variable and its facets were the most effective predictors of success criteria. In contrast, Smithers et al. [5] found that conscientiousness was not associated with academic performance, but openness to experience was related to clinic success.

Numerous previous studies have contributed to investigating the validity of selection measures in dental education. However, several aspects of these studies are worth noting. Some studies [2–5] used Costa and McCrae's NEO-PI-R and NEO-PI-3 to assess the personality of dental students. These measures are enormous in their structures, composed of 240 items measuring thirty traits of the significant five factors of a personality. Virtue et al. [6] and Patterson et al. [7] have highlighted the challenges to finding psychometrically robust selection methods. Thus, Smithers et al. [5] and Gafni et al. [8] recommended that a personality measure used in dental research be first psychometrically sound and valid.

Success in A-levels was thought to correlate well with preclinical years but less well during the clinical curriculum. However, Montague & Odds [9] suggested that A-level chemistry results correlate well throughout the undergraduate medical course. In addition to intellectual ability, personality and motivation also play a part in success in medical school performance, and these two factors are reported to account for another 40% of the variance [1]. Traditionally, the interview has been used to measure these, but the reliability of interviews has not been impressive, nor has their predictive ability.

A variety of personality tests have been used over the years in different parts of the world. Although some have been shown to have a value, particularly in predicting clinical competence [10], others have not [11, 12].

Research has supported students' personality as an essential factor for success either in their academic or professional lives [6, 13–18]. However, the studies on personality have been very few in medical education [7] and rare in dental education [19]. Thus, more research within this line is still wanted.

Empirical evidence has demonstrated that conscientiousness is the most robust and most consistent Big Five personality trait for predicting achievement outcomes (mostly, grade point average (GPA) [20–22]. Likewise, dental education studies have provided similar evidence that conscientious dental students reach high academic and clinical performance [2–4]). Therefore, this study is aimed at examining the relationships between academic performance and personality and investigating if personality factors could be used to predict the success/failure of dental students.

## 2. Material and Methods

### 2.1. Study Context

College of Dentistry, Ajman University, is a private dental college with a 5-year curriculum and offers 190 places to incoming students annually. In the first 2 years of the undergraduate program, courses are predominantly introductory and nonclinical courses, while in the subsequent years, students have clinical courses. UAE universities accept students directly from high schools. Admission to dental school is a very competitive process and depends on the student's high school grades, English language proficiency, and interview [23].

Each clinical course required completing a minimum number of defined treatment procedures. The attending faculty awarded each successful clinical procedure a quality grade. These quality grades were used with a quantitative assessment to determine the final course grade. Final grades were averaged for each student in the following clinical courses: Year 4: Endodontics, Periodontics, Restorative Dentistry, Prosthodontics, Oral Surgery, Oral Medicine, Orthodontics, and Pediatric Dentistry; 5^th^ Year: Clinical dentistry I & II (comprehensive patient management).

### 2.2. Study Participants

The study includes all fifth-year dental students registered at the College of Dentistry, Ajman University, in 2019/2020. Of the 170 participants, 60% were female and 40% were male. Participants ranged in age from twenty-four to twenty-seven years, with an average age of twenty-four years. The participants represent different nationalities, with 95% of the total coming from Arab nations. Ethical approval for the study was obtained from an ethics committee of Ajman University on 18.05.2019, Reference Number: D-F-H-19-04-29.

### 2.3. Measures of Prediction

#### 2.3.1. Big Five Inventory (BFI)

Big Five Inventory (BFI), designed as a short instrument, was used to measure the Big Five Factors of personality [24]. Using a 5-point Likert scale, the participants respond to 44 BFI items. Factors, number of items, and item examples are as follows: (a) extroversion (8 items; “I see myself as someone talkative.”), (b) conscientiousness (9 items; “I see myself as someone who does a thorough job.”), (c) neuroticism (8 items; “I see myself as someone who is depressed, blue.”), (d) openness (10 items; “I see myself as someone original, comes up with new ideas.”), and (e) agreeableness (9 items; “I see myself as someone who tends to find fault with others.”).

In this study, BFI was used to measure the convergent and discriminate validity of the conscientiousness measures. Three points need to be mentioned justifying the BFI selection. First, the instrument has been proved to be cross-culture valid. In his attempt to validate the BFI-Arabic version on the nonclinical sample, Al Ansari's study (2016) showed acceptable data about its reliability and validity. Similar results were evident in the Turkish university student sample [25]. Second, John and Srivastava [26] found that convergence between the BFI and the NEO-FFI (short version of NEO inventory) was substantial (mean = 0.93), suggesting that the conceptualizations of the five factors are almost entirely equivalent across these instruments. Third, the previous studies showed that the BFI is an effective way to be applied in the structural analyses for models of conscientiousness facets see MacCann, Duckworth and Roberts [27]. In summary, the selection of FBI is based on its cross-culture validity, its structure to a certain degree equivalent to NEO scales, and effective use in structural analyses.

#### 2.3.2. Conscientiousness NEO-PI-R Scales

Costa and McCrae's conscientiousness NEO-PI-R [28] was used to assess the facets of conscientiousness. This measure has 48 items that were specifically designed to assess the six facets of the conscientiousness factor of personality: competence (CO) (8 items; “I am efficient and effective at my work.”), order (O) (8 items; “I keep my belongings neat and clean.”), dutifulness (DU) (8 items; “I try to do jobs carefully, so they will not have to be done again.”), achievement (A) (8 items; “I strive to achieve all I can.”), self-discipline (S) (8 items; “I have trouble making myself do what I should.”), and deliberation (DE) (8 items; “I rarely make a hasty decision.”).

### 2.4. Measures of Performance

#### 2.4.1. Weighted GPA

The first criterion of success was a weighted grade point average (GPA) of academic courses for four years of dental study. This academic measure reflects basic knowledge of the dental curriculum. Weighted GPA was a composite measure of average derived from the forty-five courses with 100 credited hours that a dental student was required to take through the first year to the fourth year, and each course was weighted by its credited hours. The authors obtained the information from the Office of Admission and Registration at Ajman University.

#### 2.4.2. Weighted GPA of the Clinical Dentistry Course

The second criterion was *a weighted GPA* of the clinical dentistry course; this is the clinical course with six credit hours and includes comprehensive dental treatment of several clinical cases and faculty in addition to the final examination, which is composed of case presentation, oral and written examination, and regularly assessed students' performance.

### 2.5. Psychometric Data of Predictors

#### 2.5.1. Reliability

The consistency of the conscientiousness domain and facet measures was examined for dental student samples. Reliabilities were expected to approximate the values obtained in the conscientiousness NEO-PI-R measures applied on nonmedical samples and documented by Costa and McCrae [28].

### 2.6. Convergent and Discriminate Validity

The discriminate and convergent validity was examined for predictors in the dental education context. Correlations between the conscientiousness facet scores in NEO-PI-R and scores on the five dimensions of the Big Five Inventory (BFI) were performed. The measures were expected to be convergently valid if the NEO-PI-R conscientiousness facets are related to the conscientiousness scores of BFI. In addition, these measures are discriminately valid if the facet scores were not related to the other broad factors of a personality [29].

#### 2.6.1. Correlations of Predictors with Success Criteria

The associations of the conscientiousness domain and its facets with weighted GPA of academic courses and weighted GPA of the clinical dentistry course were examined. To examine the predictive validity of the conscientiousness factor and its six facets, one hundred and seventy dental students were selected; the ratio of participants to predictor was approximately 25 : 1.

#### 2.6.2. Incremental Validity of the Traits of Conscientiousness

Incremental validity is an important index to estimate the individual amount of validity when two or more predictors are used simultaneously [30]. Typically, it estimates the incremental prediction of facets over factors by subtracting the variance explained in a criterion by factors from that explained by facets [31]. The third goal in this study was to examine the value of conscientiousness' narrow traits over global conscientiousness in predicting college performance. The issue is whether the use of facets of conscientiousness in prediction may be more beneficial than the domain. Hierarchical regressions were used to assess the incremental validity of the traits of conscientiousness over the global conscientiousness across two performance criteria, academic and clinic. Performance was regressed onto global conscientiousness (Step 1) followed by the six narrow traits: achievement, competence, order, dutifulness, self-discipline, and deliberation (Step 2).

## 3. Results

### 3.1. Reliability and Construct Validity of Broad Conscientiousness

To assess the effectiveness of the NEO-IP-R conscientiousness, reliability and construct validity indices were explored on a dental student sample.

#### 3.1.1. Reliability

Overall, Table 1 shows that the coefficient alpha reliabilities for the broad and narrow scales of the NEO-IP-R conscientiousness were impressive. All scales demonstrated acceptable levels of reliability with a mean of 0.78. Across the scales, competence, dutifulness, and achievement were measured most reliably (all clearly above the level of 0.80), whereas order, self-discipline, and deliberation tended to be somewhat less reliable. The broad conscientiousness has recorded a high level of alpha index, with 0.90. This result is relatively similar to the level of reliability of BFI conscientiousness with 0.85 [24]. Except for the extraversion scale, neuroticism, openness, and agreeableness have recorded acceptable levels of reliabilities.

#### 3.1.2. Convergent and Discriminate Validity of the Broad Conscientiousness

We assume that the measure of broad conscientiousness has convergent validity if it is highly correlated with the scores of other measures of the same variable and has discriminate validity if it has a low correlation with variables assuming theoretically unrelated to it. The results from Table 2 shows that the convergent validity of the broad conscientiousness is evident; there is a strong positive correlation between the two broad measures of conscientiousness, with 0.85. Moreover, discriminate correlations are low, with an average of 0.34 and a standard deviation of 0.03. Agreeableness showed the strongest association with broad conscientiousness (*r* = 0.38). None of the discriminate correlations reached 0.40. In general, broad conscientiousness has convergent and discriminate validity based on the dental student sample.

### 3.2. Convergent and Discriminate Validity of the Facets of Conscientiousness

#### 3.2.1. Assumptions of Convergent and Discriminate Validity of the Facets of Conscientiousness

Three patterns of correlations were examined to assess the convergent and discriminate validity of the facets of conscientiousness. We assume there is convergent validity if the intercorrelations between facets are first at a level of approximately 0.50. Second, because there is a high common variance between conscientiousness and its traits, it is expected to have high correlations between the traits of conscientiousness and the two broad measures of conscientiousness, BFI and NEO conscientiousness. For discriminate validity, we assume low correlations between the narrow traits and the other BFI measures, neuroticism, extraversion, openness, and agreeableness, theoretically unrelated to conscientiousness.

#### 3.2.2. Convergent Validity of the Facets of Conscientiousness

Table 3 shows statistically significant, moderately strong associations between the facets of conscientiousness. Correlations ranged from 0.27 (between order and deliberation) to 0.69 (between competence and achievement), with mean (*M* = 0.49) and standard deviation (SD = 0.12). This level of meaning is an indication of convergent validity. Table 3 also presents correlations between the six facets and broad measures of conscientiousness (NEO-PI-R and BFI). The NEO-PI-R conscientiousness correlations with the facets ranged from 0.60 (dutifulness) to 0.80 (self-discipline), with a mean (*M* = 0.71) and standard deviation (SD = 0.07). For the BFI measure of conscientiousness, the range of correlations is between 0.49 (deliberation) and 0.76 (self-discipline), with a mean (*M* = 0.64) and standard deviation (SD = 0.1). Both measures of conscientiousness provide additional evidence that the facets of conscientiousness have convergent validity.

#### 3.2.3. Discriminate Validity of the Facets of Conscientiousness

Table 4 presents a pattern of correlations between conscientiousness traits and BFI measures. The BFI domain correlations with the facets ranged from 0.60 (dutifulness) to 0.80 (self-discipline), with a mean (*M* = 0.71) and standard deviation (SD = 0.07). Only three out of 24 measures record high correlations, more than 0.40. This result indicates that the traits of conscientiousness have discriminate validity.

#### 3.2.4. Predictive Validity of Domain and Facets of Conscientiousness Measures

Table 5 shows that conscientiousness recorded the strongest association with the academic and clinical performance of dentistry students compared to the other BF model domains; conscientiousness predicted GPA (*r* = 0.30, *p* < 0.01) and clinical marks (*r* = 0.31, *p* < 0.01). More conscientious students performed better in academic and clinic courses.

#### 3.2.5. Predictive Validity of Conscientiousness and Its Facets on Performance Criteria

To identify the conscientiousness facets predicting academic and clinical performance in dental education, Table 6 shows that four conscientiousness facets were strongly associated with AGPA at the *p* = 0.01 level of significance: competence (*r* = 0.33), dutifulness (*r* = 0.35), achievement (*r* = 0.30), and self-discipline (*r* = 0.23). The order facet was moderately associated at the *p* = 0.05 level of significance, *r* = 0.16. In the different patterns of associations, clinical work was strongly associated with dutifulness (*r* = 0.35, *p* < 0.01), while it was moderately associated with achievement (*r* = 0.18, *p* < 0.05) and competence (*r* = 0.19, *p* < 0.05).

#### 3.2.6. Incremental Validity of Conscientiousness Measures

To assess the incremental validity of the traits of conscientiousness over the global conscientiousness. The primary issue is whether a narrow trait of conscientiousness accounts for variance in performance criteria beyond that accounted for by the broad measure of conscientiousness. As a method of testing for incremental validity of conscientiousness' narrow traits over global conscientiousness in predicting academic and clinical performance, hierarchical regression was used. Performance was regressed onto global conscientiousness (Step 1) followed by the six narrow traits: achievement, competence, order, dutifulness, self-discipline, and deliberation (Step 2).  Table 7 presents the results from these regression analyses. A separate regression analysis was performed for each of the two college performance criteria.

The results suggest that the degree to which narrow traits contribute to predicting performance above global conscientiousness depends on the type of performance criterion. The regression analyses indicated statistically significant increases in explained variance above global conscientiousness across all performance criteria. For academic performance, the variance explained by the narrow traits over global conscientiousness was substantial (**Ѧ** **R**^2^ = 0.15), as was the percentage of variance in clinical performance explained by the narrow traits above global conscientiousness (**Ѧ** **R**^2^ = 0.10). In particular, dutifulness appeared to be the optimal predictor of academic and clinical performances, demonstrating a considerable beta weight of 0.37 for academic and 0.27 for the clinic. Self-discipline and deliberation traits were observed to contribute to predicting academic performance. Thus, it seems that some narrow traits contribute substantially to the prediction of performance criteria but not others.

## 4. Discussion

This study developed an expectation that the inclusion of only the personality variable of conscientiousness in student selection processes leads to a better prediction of success criteria in dental education and would reduce the negative impact due to the current practices in personality assessment. In addition, the study adopted the NEO-PI-R conscientiousness scales and made an expectation that they are reliable and valid selection tools in assessing conscientiousness and its specific traits for dental candidates and students.

To evaluate these expectations, the study first examined psychometric characteristics of the conscientiousness scales, including reliabilities and construct validity. The study presented encouraging evidence on the conscientiousness scales within the dental education context, demonstrating their robust psychometric characteristics. The reliabilities for the broad factor and its six traits were impressive, showing acceptable levels (internal consistencies were higher than 0.70, Nunnaly's criterion [32], and inconsistent with those indices recorded on nonmedical student samples by MacCann, Duckworth, and Roberts [27] and by Madhavan [33] when evaluating the nine facets of conscientiousness from the IPIP Big Five scales. As to convergent validity, the broad measure of conscientiousness was highly correlated with their facets. Regarding discriminant validity, the conscientiousness facets showed moderate to weak overlap with neuroticism, extraversion, openness to experience, and agreeableness.

The second step was to investigate whether the conscientiousness scales could predict performances desired in dental schools. The relationships were evaluated on two levels: the broad dimension of conscientiousness and the lower-order traits. The variations among relationships of personality factors with success criteria were evident broadly. However, conscientiousness was the best predictor for academic education and clinical training success. With more minor degrees, agreeableness was also correlated with success criteria. Extraversion and openness were significantly related to academic achievement but not clinical performance. Neuroticism did not predict any of the two criterion variables. Our results are consistent with much of the previous evidence that conscientiousness was the best predictor of academic performance [3].

Another pattern of associations between personality and academic achievement and clinical performance was presented in the specific conscientiousness scales. Competence, dutifulness, and achievement were strongly related to the success criteria, and order and self-discipline predicted academic achievement but did not predict the clinic performance. These results are consistent with the previous research that confirmed the power of competence and dutifulness facets in predicting academic and clinical performance [2, 4]. Our study added new predictors of conscientiousness to the success in dental schools by focusing on the role of achievement and self-discipline in predicting academic performance. However, there was a divergence between our results and previous research regarding deliberation. In our study, deliberation was uncorrelated with any of the criterion variables, and this variable was found to be correlated with the academic coursework [2] and was a significant predictor for clinical performance [4].

In addition to the evidence that the broad measure of conscientiousness is necessary, the prediction accuracy is improved by including its narrow traits. This study further confirmed a role for the narrow traits of conscientiousness as predictors of performance. However, a final determination of the narrow traits that substantially contribute to the prediction requires further investigation. Our study found that the dutifulness trait improved academic and clinical performance prediction. Additionally, other traits of self-discipline and deliberation were identified to be helpful in the prediction of academic performance. Previous studies were not consistent regarding these results. Two previous studies have been interested in this line, and they have shown variation in which trait of conscientiousness contributes to the prediction. Chamberlain, Catano, and Cunningham [2] found significant correlations between competence and third-year clinical and academic performance, while dutifulness and deliberation traits correlate with academic performance. The facet associations were observed in the Stacey and Kurunathan study [4]. All facets of conscientiousness correlated with clinical points rated by instructors. Except for the deliberation trait, the results showed similar associations with the other facets' academic performance in selected subjects. In regression analyses, the dutifulness facet combined with certain facets of other domains had explained a moderate percentage of the variance of the academic performance; likewise, the deliberation trait combined with other certain facets of other domains explained a moderate percentage of the variance of the clinical performance.

In general, the study had developed an expectation that broad conscientiousness and its narrow traits of order, achievement, dutifulness, deliberation, competence, and self-discipline would predict success in dental schools. This was the case to broad conscientiousness, competence, achievement, and dutifulness. To some extent, order and self-discipline only succeeded in predicting academic performance, and however, deliberation failed to predict any of the success criteria. The conscientiousness results indicate that students who feel well prepared to deal with life, work hard to achieve their goals, and scrupulously fulfil their moral obligations stable and well-organized performed better in academic work. However, the same results raise questions about the model of current clinical training, and they suggest that students who were less emotionally stable, less organized, and did not think carefully before they acted received higher grades in their clinical studies.

Some researchers indicated that personality factors play an insignificant part in success or failure, and other more relevant factors were identified [34–36] have argued that although there are some personality differences between dental students and the different types of dental practice to which they are subsequently drawn, personality tests do not discriminate reliably between specialty groups. It would also appear from this investigation that these tests do not differentiate between students with problems and those without. A-level point scores, which have traditionally been one of the significant criteria based on student selection, also bore no relationship to eventual academic performance. Indeed, those students who obtained a degree before entering dental school had poorer A-level points than others. This supports the hypothesis that motivation plays an important part in success during a dental undergraduate's career since such students may have many distractions (such as financial worries).

Most dental studies have been limited to investigating the power of cognitive abilities and personality traits in predicting success. Further research is needed to explore how conscientiousness characteristics for students interact with study behaviours to produce successful performance in academic and clinical training.

This study and other previous studies on personality predicting performance in dental schools were limited to two criteria of performance GPA and clinical training marks. However, research references other performance outcomes beyond GPA, such as leadership, interpersonal skills, physical and psychological health, career orientation, adaptability, and perseverance [37]. Dental schools reflect the importance of these outcomes as indicators of success in school mission statements. We need further studies focusing on the prediction power of conscientiousness and its traits on alternative academic performance indicators. We may better understand how each facet of conscientiousness impacts essential outcomes. For example, Mcabee, Oswald, and Connelly [21], using HEXACO conscientiousness measures, found that diligence predicted perseverance, continuous learning, and social responsibility. Perfectionism negatively predicted students' physical and psychological health and adaptability. With accumulating knowledge about facets and their correlates, administrators may base their selection decisions on facets of conscientiousness.

Despite these encouraging findings, this study has several limitations. First, this study involved a relatively small number of students that might compromise the accuracy of the results obtained from the analysis. Second, this study was confined to one cohort of dental students of one dental college and in one country, limiting the generalizability of its results. Lastly, personality measurement was collected through the face-to-face method, which was not entirely anonymous and may lead to response bias. Despite these limitations, this study has several strengths. First, a prospective study design was used, which was able to explore the relationship between students' personalities and academic performance. Secondly, the test we used has robust measures of the facets of conscientiousness that are sufficiently reliable and possess a high level of convergent and discriminate validity. Considering the limitations and strengths, the results of this study should be interpreted with caution and within its context.

## 5. Conclusions and Future Directions

Our results revealed that it is possible to obtain robust measures of the facets of conscientiousness that are sufficiently reliable and possess a high level of convergent and discriminate validity.

This study confirms that the students' personality profile is a substantial predictor of academic performance and likely to help select future intakes of students. However, a prospective study with a larger sample size among multicenter would be required for a definite answer to this question.

## Figures and Tables

**Table 1:** Alpha coefficient reliabilities of NEO-IP-R conscientiousness scales.

Scales	Number of items in scale	Mean	Standard deviation	Alpha
NEO-IP-R conscientiousness scales				
Competence	8	22.18	4.87	0.83
Order	8	20.30	5.70	0.72
Dutifulness	8	24.70	4.48	0.87
Achievement	8	21.96	6.25	0.83
Self-discipline	8	19.23	5.53	0.72
Deliberation	8	18.27	4.99	0.71
Broad conscientiousness	48	112.5	23.2	0.90
BFI scales				
Neuroticism	9	26.75	6.40	0.74
Extraversion	8	18.50	4.88	0.64
Openness	10	32.38	7.55	0.78
Agreeableness	8	30.96	9.20	0.87
Conscientiousness	9	28.58	8.32	0.85

**Table 2:** Convergent and discriminate coefficients of conscientiousness.

BFI scales	NEO conscientiousness
Conscientiousness	0.85^∗∗^
Neuroticism	-0.32^∗∗^
Extraversion	0.32^∗∗^
Openness	0.34^∗∗^
Agreeableness	0.38^∗∗^

^∗∗^ *p* < 0.01 (*N* = 144).

**Table 3:** Convergent coefficients of facets of conscientiousness.

	1	2	3	4	5	6
(1) Competence	1					
(2) Order	0.38^∗∗^	1				
(3) Dutifulness	0.47^∗∗^	0.35^∗∗^	1			
(4) Achievement	0.69^∗∗^	0.48^∗∗^	0.55^∗∗^	1		
(5) Self-discipline	0.62^∗∗^	0.55^∗∗^	0.59^∗∗^	0.67^∗∗^	1	
(6) Deliberation	0.50^∗∗^	0.27^∗^	0.41^∗∗^	0.38^∗∗^	0.48^∗∗^	1
NEO conscientiousness	0.73^∗∗^	0.76^∗∗^	0.60^∗∗^	0.70^∗∗^	0.80^∗∗^	0.66^∗∗^
BFI conscientiousness	0.68^∗∗^	0.65^∗∗^	0.57^∗∗^	0.71^∗∗^	0.76^∗∗^	0.49^∗∗^

^∗^ *p* < 0.05,  ^∗∗^*p* < 0.01 (*N* = 144).

**Table 4:** Discriminate coefficients of facets of conscientiousness.

BFI measures	Competence	Order	Dutifulness	Achievement	Self-discipline	Deliberation
Neuroticism	-0.38^∗∗^	-0.18	-0.03	-0.35^∗∗^	-0.29^∗∗^	-0.27^∗∗^
Extraversion	0.44^∗∗^	0.22^∗^	0.21^∗^	0.50^∗∗^	0.37^∗∗^	0.03
Openness	0.39^∗∗^	0.20	0.35^∗∗^	0.40^∗∗^	0.47^∗∗^	0.33^∗∗^
Agreeableness	0.19	0.29^∗∗^	0.38^∗∗^	0.15	0.21^∗^	0.25^∗^

^∗^ *p* < 0.05,  ^∗∗^*p* < 0.01 (*N* = 144).

**Table 5:** Correlations of BFI scales with performance criteria.

BFI scales	GPA	Clinic
Neuroticism	-0.06	-0.02
Extraversion	0.14^∗^	0.03
Openness	0.15^∗^	0.02
Agreeableness	0.21^∗∗^	0.30^∗∗^
Conscientiousness	0.30^∗∗^	0.31^∗∗^

**Table 6:** Correlations of conscientiousness with performance criteria.

NEO-IP-R scales	GPA	Clinic	NEO-IP-R scales	GPA	Clinic
Competence	0.33^∗∗^	0.19^∗^	Achievement	0.30^∗∗^	0.18^∗^
Order	0.16^∗^	0.08	Self-discipline	0.23^∗∗^	0.09
Dutifulness	0.35^∗∗^	0.31^∗∗^	Deliberation	0.12	0.06
Conscientiousness					

^∗^ *p* < 0.05,  ^∗∗^*p* < 0.01 (*N* = 144).

**Table 7:** Hierarchical regression results for performance criterion.

	**B**	**R** ^2^	**Ѧ** **R**^2^
Academic performance			
(1) Global conscientiousness	0.30	0.07	
(2) Global conscientiousness	0.22	0.22	0.15
(3) Dutifulness	0.38^∗∗^		
(4) Self-discipline	-31^∗∗^		
(5) Deliberation	-0.21^∗^		
Clinic performance			
(1) Global conscientiousness	0.21	0.04	
(2) Global conscientiousness	0.31	0.14	0.10
(3) Dutifulness	0.27^∗∗^		

Numbers 1 and 2 indicate Step 1 and Step 2 of the hierarchical regression analyses. ^∗^*p* < 0.05,  ^∗∗^*p* < 0.01 (*N* = 144).

## Data Availability

Any data related to the study can be readily provided on reasonable request.
